# Clinical and genetic heterogeneity of adult polyglucosan body disease caused by *GBE1* biallelic mutations in China

**DOI:** 10.1016/j.gendis.2023.101140

**Published:** 2023-10-16

**Authors:** Yikun Chen, Yan Shi, Yuan Gao, Yan Hu, Linying Zhou, Jingmei Hong, Shirui Gan, Xiang Lin, Wanjin Chen, Guorong Xu, Jin He

**Affiliations:** a Department of Neurology and Institute of Neurology of First Affiliated Hospital, Institute of Neuroscience, and Fujian Key Laboratory of Molecular Neurology, Fujian Medical University, Fuzhou, Fujian 350005, China; b Fujian Key Laboratory of Molecular Neurology, Institute of Neuroscience, Fujian Medical University, Fuzhou, Fujian 350005, China; c Electron Microscopy Lab of Public Technology Service Center, Fujian Medical University, Fuzhou, Fujian 350005, China

Adult polyglucosan body disease (APBD) is a rare and highly heterogeneous glycogen storage disorder due to biallelic variants in *GBE1*.^1^ Typical APBD presentations include gait abnormalities with polyneuropathy, leukodystrophy, neurogenic bladder, and mild cognitive impairment. Differential diagnosis of APBD encompasses a large spectrum of conditions including axonal and demyelinating sensorimotor polyneuropathy, progressive spastic paraparesis, and leukodystrophies. The majority of APBD patients are Ashkenazi-Jewish harboring a homozygous *GBE1* mutation, c.986A > C. There have been a few APBD patients reported in East Asia. In addition, the large deletion mutations of *GBE1* have only been previously described in early congenital neuromuscular patients.^2^ In this study, we depicted the phenotypic heterogeneity in four APBD patients in China and identified five single nucleotide variants and two large deletion variants in *GBE1.* These findings expand the clinical and genetic spectrum of APBD patients and reaffirm the importance of a more comprehensive and cautious diagnostic approach.

By analyzing whole-exome sequencing data of 555 gait disorder patients including 121 patients from the Charcot-Marie-Tooth Disease cohort (NCT04010188), 153 patients from the Cerebellar Ataxia cohort (NCT04010214), and 281 patients from Spastic Paraplegia cohort (NCT04006418), we identified four patients harboring variants in *GBE1* gene which highly implied the disease-causing relation. In these four patients we identified seven variants (five missense variants and two deletion variants) in *GBE1* gene (NM_000158.4): exon 7 deletion and c.466C > T in patient 1, exon 3–7 and c.466C > T in patient 2, c.610G > T and c.1627T > G in patient 3, and c.1612T > G and c.1760T > A in patient 4 (Fig. 1A–D). Pathogenicity analysis of GBE1 variants is summarized in Table S1. For patient 1, we initially only detected a heterozygous c.466C > T variant which presented a low frequency in databases and was predicted to be deleterious by *in silico* software. Of note, in patient 2, we identified the same variant but it was manifested as homozygous in VCF file and Sanger sequencing. Upon careful reexamination of the BAM file, we found the average sequencing depth of the *GBE1* gene was lower than controls. Inspired by previous reports about the copy number variants in *GBE1* and similar clinical presentation among these patients, we assumed that the c.466C > T variant might be pathogenic and these two patients might harbor potential copy number variants. To discover this, we performed whole-genome sequencing on patients 1 and 2. Focus on the *GBE1* region, we rapidly identified a 16 kBP deletion (range chr3:81676420-81692774, covering exon 7) and a 74 kBP deletion (range chr3: 81676183–81750802, covering exon 3 to 7) in patient 1 and patient 2, respectively (Fig. 1E, F). Then breakpoints were confirmed both in gDNA and cDNA level with Sanger sequencing. Then we identified two kinds of microhomology around the two breakpoint junctions, implying the deletion may be mediated by nonhomologous DNA end-joining (Fig. 1G, H).^3^ Congregation analysis for all candidate variants on all patients and available family member samples were conducted (Fig. S1A–F).

**Figure 1:**
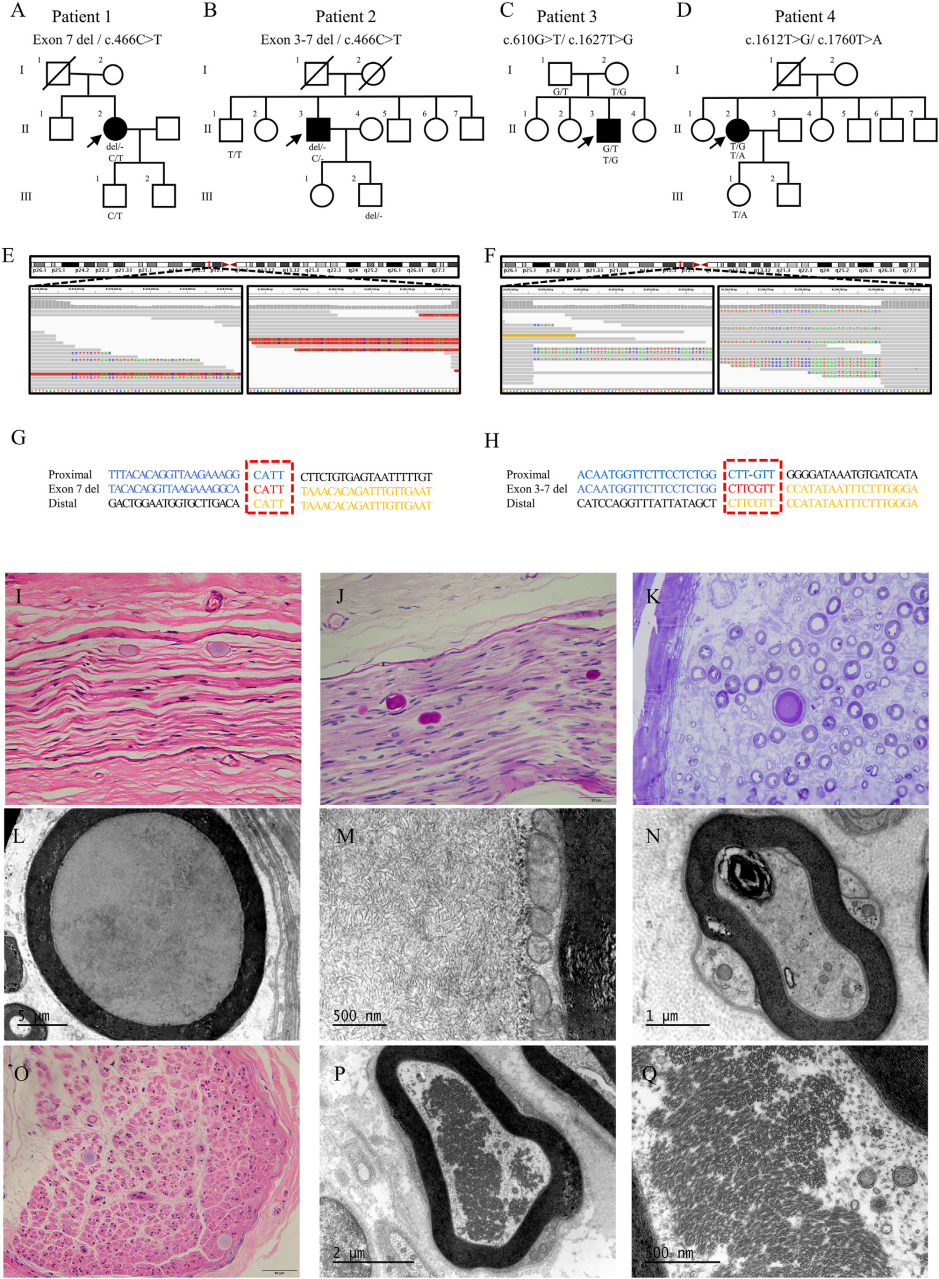
Genetic and pathological findings in adult polyglucosan body disease (APBD) patients. **(A**–**D)** Pedigree of four APBD patients. The squares represent males and the circles females. The filled black boxes indicate patients and the white boxes unaffected relatives. The arrow indicates the proband. **(E, F)** The integrative genomics viewer images of the whole genome sequencing (WGS) read alignments near the breakpoints of patients 1 and 2 respectively. Soft-clipped reads show mismatched or unaligned bases. **(G, H)** Junction sequences near breakpoints of patients 1 and 2. DNA sequences obtained from Sanger sequencing were aligned to the proximal and distal reference sequences. The presence of microhomology is indicated by the red dashed box. **(I, O)** Hematoxylin and eosin staining showed large PBs in neuronal axons of patients 1 and 3, respectively. **(J, K)** Periodic acid-Schiff staining showed a positive reaction of PBs and toluidine blue staining also revealed loss of myelinated fibers and regeneration cluster of thinly myelinated fibers. **(L**–**N)** Transmission electron micrographs of patient 1 showed irregularly branched filaments of PB and squeezed mitochondria around the myelin sheath lamellar. Another myelinated axon showed swollen mitochondria and demyelinating changes. The osmiophilic precipitate was likely associated with degeneration and autolysis. **(P, Q)** Transmission electron micrographs of patient 3 revealed that filaments were loosely packed, with small dense cores deposited within the myelin sheath, swollen mitochondria, and demyelination. Scale bar is 50 μm for (I, J, O), 20 μm for (K), 5 μm for (L), 2 μm for (P), 1 μm for (N), and 500 nm for (M, Q).

To better describe the manifestations of the four ABPD patients, we fully as possible collected the clinical data including neurological examination, electrophysiological study, and craniocerebral and spinal magnetic resonance imaging. These patients shared similar clinical characteristics including late-onset discomfort in lower limbs with a mean age of 55.5 ± 4.97 years and abnormal magnetic resonance imaging findings like leukodystrophies, mild to prominent cerebral and cerebellar atrophy, and thoracic cord atrophy (Fig. S2). However, they still showed great heterogeneity in onset symptoms and clinical signs. Three patients except patient 4 demonstrated widespread neurogenic damage. Patient 1 also presented predominant signs of ataxia while patients 2 and 3 demonstrated more numbness. Patient 4 showed increased muscle tone with unremarkable neurophysiological results. The clinical, magnetic resonance imaging, and electrophysiological findings and primary diagnosis of the four patients are summarized in Table S2.

In addition, patients 1, 3, and 4 underwent distal sural nerve biopsy, which showed typical histopathological features under light microscope. The hematoxylin and eosin staining revealed basophilic intracytoplasmic polyglucosan bodies (PBs) within the axonal region of nerve tubes in all patients. Patient 1 also showed inclusions positive for periodic acid-Schiff staining with amylopectin-like material, which is a typical feature of PBs. The toluidine blue staining of patient 1 showed a large intra-axonal PB, loss of myelinated fibers, and a number of regeneration clusters of thinly myelinated fibers (Fig. 1I, J, O, M; Fig. S1G). The PBs demonstrated different morphology at the ultrastructural level. Transmission electron microscopy revealed that the PB in patient 1 was about 20 μm in diameter and consisted of irregular fine filaments. The mitochondria were squeezed to edge around the myelin sheath lamellar by the enlarging PBs. Another myelinated axon showed swollen mitochondria, disappeared cristae, and demyelinating changes under electron microscopy. The osmiophilic precipitate was considered associated with degeneration and autolysis. Sections from patient 3 showed filaments loosely packed, with small dense cores deposited within the myelin sheath, swollen mitochondria, and demyelination (Fig. 1L–N, P, Q).

The later-onset and complex phenotype of APBD may delay the real diagnosis by approximately five years, with misdiagnosis including cerebral small vessel disease, multiple sclerosis, and amyotrophic lateral sclerosis.^4^ In this study, patients were initially diagnosed with spinocerebellar ataxia, peripheral neuropathy, and spastic paraplegia respectively due to their predominant symptoms. Although the clinical features of these patients were shared in previous reports, it was still challenging for clinicians to provide a timely and accurate diagnosis. Molecular genetic testing is currently recommended as a first step for diagnosis. In this study, we identified two large deletion mutations of *GBE1* in APBD patients for the first time which are hard to be detected by whole-exome sequencing. We briefly reviewed these reported patients with deletion variants (Table S3) and found that these patients were all early congenital neuromuscular type either harboring homozygous exon deletions or compound heterozygous mutations with another truncated mutation. So, we assumed that all missense variants harbored by patients might retain certain enzyme activity to contribute to the APBD phenotype. While more deleterious mutation combinations of *GEB1* would result in Andersen's disease, complete loss of enzyme activity is not compatible with life. Declined enzyme activity due to *GBE1* gene variants results in less branched and soluble glycogen, which tends to aggregate and form PBs that deposit in tissues. However, PBs have been reported in other conditions including Lafora disease, diabetic neuropathy, motor neuron disease, and even in elderly people without neurologic disease.^5^ Our sural nerve biopsy disclosed typical PBs and characterized the pathological changes at the ultrastructure level, pathologically confirming the diagnosis of APBD. Hence this study further highlights the significance of awareness and early correct diagnosis of APBD and proposes a comprehensive and cautious diagnostic approach combined with whole-genome sequencing and sural nerve biopsy if necessary.

In conclusion, we identified seven pathogenic variants in *GBE1* and described the detailed clinical demonstration of APBD patients in China. Our study expanded the clinical and genetic spectrum of this disease and emphasized the necessity to consider copy number mutations in *GBE1*.

## Author contributions

Conception and design of the study: J.H., G.R.X.; acquisition and analysis of data: Y.K.C., Y.S., Y.G., Y.H., L.Y.Z., J.M.H., S.R.G., X.L., W.J.C.; manuscript drafting: Y.K.C., J.H.; figure preparation: Y.K.C., J.H.

## Conflict of interests

The authors declare no conflict of interests exists.

## Funding

This work was supported by grants from the National Natural Science Foundation of China (No. 82271412 to J.H., 82025012 to W.J.C., U1905210 to W.J.C.).

## Figures and Tables

**Figure S1:**
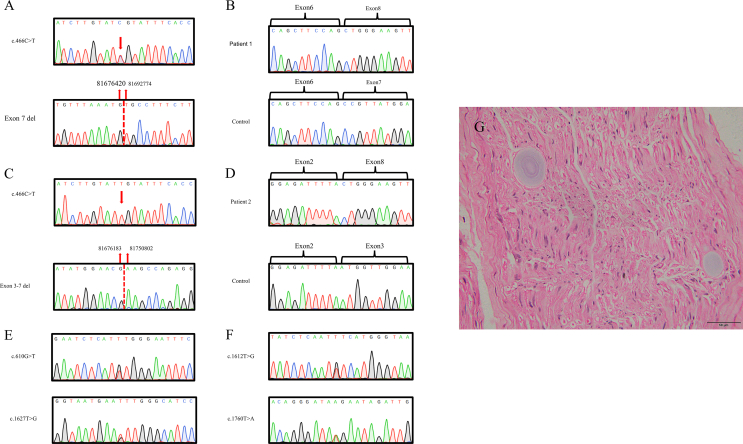
Sanger sequencing confirmation of all mutations of all patients and hematoxylin and eosin staining of patient 4. **(A, C, E, F)** Sanger sequencing confirmation of *GBE1* variants of patients 1, 2, 3, and 4 at the gDNA level. **(B, D)** Sanger sequencing confirmation of large deletion variants of GBE1 in patients 1 and 2. **(G)** Hematoxylin and eosin staining showed large PBs in the neuronal axons of patient 4.

**Figure S2:**
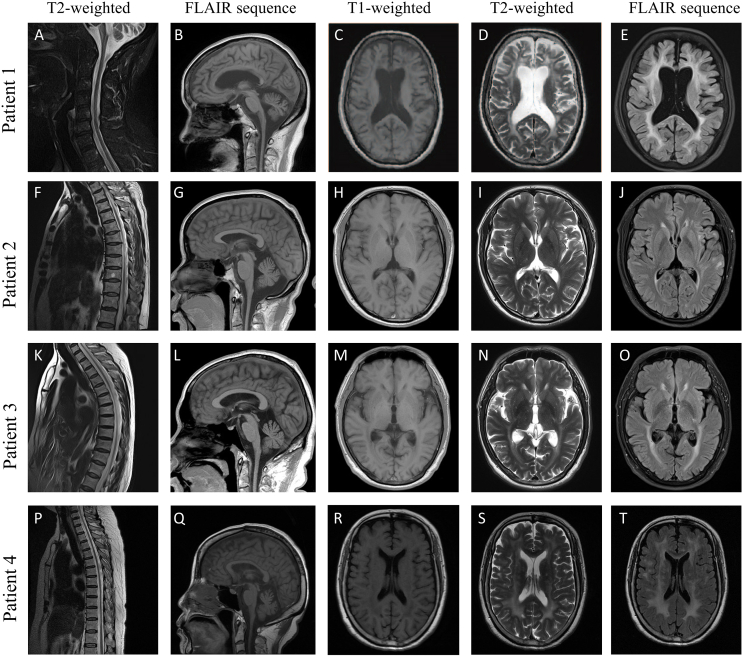
Magnetic resonance imaging of four APBD patients. **(A, B, F, G, K, L, P, Q)** Sagittal T2-weighted images showed atrophy of the spinal cord, medulla oblongata, and cerebellum. **(C–E, H–J, M–O, R–T)** Axial T1-weighted, T2-weighted, and FLAIR sequences images showed symmetric hyperintense white matter lesions in the periventricular regions.
