# Supplementary material for: Clinical and genetic heterogeneity of adult polyglucosan body disease caused by GBE1 biallelic mutations in China
Source: Genes Dis. 2023 Oct 16;11(5):101140. doi: 10.1016/j.gendis.2023.101140 (PMC11099309; doi:10.1016/j.gendis.2023.101140)
Supplement: Multimedia component 1 [file mmc1.docx]

Table S1: Pathogenicity analysis of variants in *GBE1* gene

| Location | Mutations | AA change | Mutation taster | SIFT | PolyPhen2 | CADD | dbSNP | ExAC（EAS) | gnomAD（EAS） | ACMG classification | Evidence of  pathogenicity | Reference |
| --- | --- | --- | --- | --- | --- | --- | --- | --- | --- | --- | --- | --- |
| Exon 4 | c.466C>T | p.R156C | DC(>0.999) | D(0) | PD(1) | 26.9 | rs763302311 | 0.000586 | 0.000407925 | Likely pathogenic | 3*PM, 1*PP | Novel |
| Exon 5 | c.610G>T | p.V204L | DC(>0.999) | D(0) | B（0.417） | 27.4 | N | N | N | Likely pathogenic | 2*PM, 2*PP | Reported |
| Exon 12 | c.1612T>G | p.F538V | DC(>0.999) | D(0) | PD(0.987) | 29.3 | N | N | N | Uncertain significance | 1*PM, 2*PP | Reported |
| Exon 13 | c.1627T>G | p.F543V | DC(>0.999) | D(0) | PD(1) | 26 | N | N | N | Uncertain significance | 1*PM, 2*PP | Novel |
| Exon 13 | c.1760T>A | p.M587K | DC(>0.999) | D(0) | PD(0.757) | 27.8 | N | N | N | Uncertain significance | 1*PM, 2*PP | Novel |
| Abbreviations: DC:disease causing; D:damaging; PD:probably damaging; B:benign; N:not available; PS:pathogenic strong; PM:pathogenic moderate; PP:pathogenic supporting | | | | | | | | | | | | |
