# Supplementary material for: Clinical and genetic heterogeneity of adult polyglucosan body disease caused by GBE1 biallelic mutations in China
Source: Genes Dis. 2023 Oct 16;11(5):101140. doi: 10.1016/j.gendis.2023.101140 (PMC11099309; doi:10.1016/j.gendis.2023.101140)
Supplement: Multimedia component 2 [file mmc2.docx]

Table S2: Clinical features of four APBD patients

|  | Patient1 | | Patient2 | | Patient3 | | Patient4 | |
| --- | --- | --- | --- | --- | --- | --- | --- | --- |
| mutation | c.466C>T | | c.466C>T | | c.610G>T | | c.1612T>G | |
|  | Exon 7 del | | Exon 3-7 del | | c.1627T>G | | c.1760T>A | |
| gender | female | | male | | male | | female | |
| Age of Onset | 55 | | 49 | | 55 | | 63 | |
| Age at last visit | 58 | | 55 | | 61 | | 68 | |
| Initial symptom | Gait disturbance | | Numbness in LLs | | Weakness and numbness in LLs | | Walking instability | |
| Primary Diagnosis | Spinocerebellar Ataxia | | Peripheral Neuropathy | | Peripheral Neuropathy | | Spastic Paraplegia | |
| LL Muscle Strength | proximal:4/4 distal:4/4 | | proximal:5/5 distal:4/4 | | proximal:4/4 distal:5/5 | | proximal:3/3 distal:1/2 | |
| LL Muscle Tone | N | | N | | N | | increased | |
| DTR | N | | absent | | N | | absent | |
| Sensory Disturbance | N | | sensory loss | | sensory loss | | sensory loss | |
| Babinski sign | + | | + | | + | | + | |
| Incontinence | + | | - | | + | | + | |
| Cognitive impairment | - | | + | | - | | - | |
| MNCV (m/s) | Median 50 | Tibialis 46.2 | Median 58.3 | Tibialis 38.8 | Median 51.6 | Tibialis 40.7 | Median ND | Tibialis 41.6 |
| CMAP (mV) | Median 12.3 | Tibialis 0.6 | Median 14.8 | Tibialis 0.7 | Median 13.8 | Tibialis 2.4 | Median ND | Tibialis 9.3 |
| SCV (m/s) | Median 62.5 | Sural 40 | Median 50.8 | Sural 45.3 | Median 41.1 | Sural 42 | Median ND | Sural 50.1 |
| SNAP (μV) | Median 22 | Sural 17 | Median 10 | Sural 2.9 | Median 13.8 | Sural 42 | Median ND | Sural 20.7 |
| F-waves(ms) | 51 | | 58.8 | | 57.5 | | ND | |
| Leukodystrophies | + | | + | | + | | + | |
| Other MRI findings | Prominent cerebral and cerebellar atrophy, enlarged lateral ventricles, thin corpus callosum | | Cerebellar atrophy, marked thoracic spinal cord atrophy | | Cerebellar atrophy, marked thoracic spinal cord | | Cerebral and cerebellar atrophy, thin corpus callosum | |
| N: normal; +: positive; -: negative; LL: lower limbs; DTR: deep tendon reflex; Proximal LL muscle strength were evaluated by muscle strength of hip flexion. Distal LL muscle strength were evaluated by ankle dorsiflexion and planter flexion. MNCV: motor nerve conduction velocity; cMAP: compound motor action potential; SCV: sensory conduction velocity, SNAP: sensory nerve action potential, ND: not done. | | | | | | | | |
