# Supplementary material for: Clinical and genetic heterogeneity of adult polyglucosan body disease caused by GBE1 biallelic mutations in China
Source: Genes Dis. 2023 Oct 16;11(5):101140. doi: 10.1016/j.gendis.2023.101140 (PMC11099309; doi:10.1016/j.gendis.2023.101140)
Supplement: Multimedia component 3 [file mmc3.docx]

| Table S3: The clinical features of genotype and phenotype of patients with GBE1 large deletion mutation. | | | | | | | | | | | | | | | |
| --- | --- | --- | --- | --- | --- | --- | --- | --- | --- | --- | --- | --- | --- | --- | --- |
| Family | Cases | Mutation type | Exon | Mutation | Protein alteration | Gender | Ethnicity | age of onset | Disease course | Myotonia | Reflex | Other complications | Deformity | Neuromuscular phenotypes | Reference |
| 1 | 1 | compound heterozygote | exon 4-6 | deletion | N | M | Italian | neonatal | 4 months | hypotonia | hyporeflexia | cardiopulmonary failure | N | Early congenital neuromuscular form | Bruno C et al^1^ |
|  | 2 |  | exon 13 | c.1774G>T | E592X |  |  |  | 4 weeks |  |  |  | equinovarus feet |  |  |
| 2 | 3 | homozygote | exon 12 | deletion | N | F | American | neonatal | 5 weeks | NP | hyporeflexia | respiratory failure | mild fixed flexion deformities at the elbows, hips, and ankles | Early congenital neuromuscular form | Tay SK et al^2^ |
| 3 | 4 | homozygote | exon 4-7 | deletion | N | F | German | neonatal | 18 weeks | hypotonia | hyporeflexia | hepatomegaly, cardiopulmonary dysfunction | minor contractures | Early congenital neuromuscular form | Nolte KW et al^3^ |
| 4 | 5 | homozygote | exon 16 | deletion | N | M | Chinese | neonatal | 37 days | hypotonia | very week reflexes | biventricular cardiac dysfunction | bilateral equivarus deformities of the feet | Early congenital neuromuscular form | Raju GP et al^4^ |
| 5 | 6 | compound heterozygote | exon 2 | c.288del A | G97Efs*46 | F | Chinese | neonatal | 73 days | NP | NP | cardiopulmonary dysfunction | N | Early congenital neuromuscular form | Li SC et al^5^ |
|  |  |  | exon 7 | deletion | N |  |  |  |  |  |  |  |  |  |  |
| 6 | 7 | compound heterozygote | exon 2 | c.288del A | G97Efs*46 | M | Chinese | neonatal | 2 months | hypotonia | NP | left ventricle hypertrophy and cardiac performance | N | Early congenital neuromuscular form | Li SC et al^5^ |
|  |  |  | exon 2-7 | deletion | N |  |  |  |  |  |  |  |  |  |  |
| 7 | 8 | compound heterozygote | exon 14 | c.1909C>T | R637X | F | American | neonatal | NP | NP | NP | NP | N | Early congenital neuromuscular form | Butler DC et al^6^ |
|  |  |  | exon 2-16 | deletion | N |  |  |  |  |  |  |  |  |  |  |
| 8 | 9 | compound heterozygote | exon 10 | c.1239del T | D413Efs*23 | NP | American | NP | NP | NP | NP | hypertrophic pediatric cardiomyopathy | NP | NP | Burstein DS et al^7^ |
|  |  |  | exon 1 | partial deletion | N |  |  |  |  |  |  |  |  |  |  |
| 9 | 10 | compound heterozygote | exon 4 | c.466C>T | R156C | F | Chinese | 55 | over 3 years | mild increased muscle tone in lower limbs | normal | leukodystrophies | N | APBD | This Study |
|  |  |  | exon 7 | deletion | N |  |  |  |  |  |  |  |  |  |  |
| 10 | 11 | compound heterozygote | exon 4 | c.466C>T | R156C | M | Chinese | 49 | over 6 years | normal | absent in DTR | leukodystrophies | N | APBD | This Study |
|  |  |  | exon 3-7 | deletion | N |  |  |  |  |  |  |  |  |  |  |

N: none; NP: not provided; DTR: DTR: deep tendon reflex

**References**

1. Bruno C, van Diggelen OP, Cassandrini D, et al. Clinical and genetic heterogeneity of branching enzyme deficiency (glycogenosis type IV). Neurology. 2004;63(6):1053-1058.

2. Tay SKH, Akman HO, Chung WK, et al. Fatal infantile neuromuscular presentation of glycogen storage disease type IV. Neuromuscular disorders: NMD. 2004;14(4):253-260.

3. Nolte KW, Janecke AR, Vorgerd M, Weis J, Schröder JM. Congenital type IV glycogenosis: the spectrum of pleomorphic polyglucosan bodies in muscle, nerve, and spinal cord with two novel mutations in the GBE1 gene. Acta Neuropathologica. 2008;116(5):491-506.

4. Raju GP, Li H-C, Bali DS, et al. A case of congenital glycogen storage disease type IV with a novel GBE1 mutation. Journal of Child Neurology. 2008;23(3):349-352.

5. Li S-C, Hwu W-L, Lin J-L, et al. Association of the congenital neuromuscular form of glycogen storage disease type IV with a large deletion and recurrent frameshift mutation. Journal of Child Neurology. 2012;27(2):204-208.

6. Butler DC, Glen WB, Schandl C, Phillips A. Glycogen Storage Disease Type IV Diagnosed at Fetal Autopsy. Pediatric and Developmental Pathology. 2020;23(4):301-305 %U <http://journals.sagepub.com/doi/10.1177/1093526619890224>.

7. Burstein DS, Gaynor JW, Griffis H, et al. Genetic Variant Burden and Adverse Outcomes in Pediatric Cardiomyopathy. Pediatric research. 2021;89(6):1470-1476 %U <https://www.ncbi.nlm.nih.gov/pmc/articles/PMC8256333/>.
